# The Prize Papers

**Published:** 1919-02-08

**Authors:** 


### "THE HOSPITAL" LEAFLET COMPETITION.

### THE PRIZE PAPERS.

### I. Public Health Work for Women.

Curiously little is known by the general public of the work undertaken by women in the public health service.

Statistics are published from time to time of the ravages made by preventible diseases, and comparisons are made between the number of lives lost on the battle-fields and the number of children's lives lost at home each year. These comparisons shock and arrest the attention for the time being, but it is scarcely an exaggeration to say that even amongst the rank and file of the medical and nursing professions their meaning is not fully realised, and but little is known of the systematic work undertaken by public health authorities to eradicate disease and so save the great wastage of infant life.

In consequence of this general vagueness a considerable difference obtains at present among the various local health authorities as to the qualifications required of women health visitors, etc. It is to be hoped, however, that as the wastage of human life from preventible causes is more fully realised, the aim of public health work will become better known, and the need of special training for those engaged on it will be more appreciated.

Apart from institutional treatment in hospitals for infectious diseases, tuberculosis sanatoria, clinics for venereal diseases, etc., public health authorities are primarily concerned in an attempt to reduce the high rate of mortality in infants and young children. To this end infant consultations are established, and home visiting of mothers and babies is undertaken by health visitors.

It is here that the need arises for educated women with specialised knowledge and training in maternity and child welfare. A memorandum issued by the Local Government Board on health visiting and infant-welfare centres states: "Experience proves that the three most useful qualifications for non-medical health visitors are those of (1) trained nurse, (2) certified midwife, (3) certificated sanitary inspector. The training of a nurse is valuable . . . but this training in nursing needs to be specialised in character."

### What is the Necessary Training?

Of the training here recommended it may be said that the amount and kind of nursing training a health visitor should possess is still a matter of dispute, but that it should have special reference to her future work seems only common sense. Children's hospital training, for instance, would appear to be of more value to the child-welfare worker than general training. Some medical officers of health are of opinion, from their experience of maternity and child-welfare work, that the trained nurse's outlook is too institutional; that she is too accustomed to disease, and has not a sufficient knowledge of what normal healthy childhood should be. And in practice there is much to be said for this opinion.

On the other hand, there are still health committees who make general-hospital training the only qualification required, even the second recommendation of the Local Government Board—a certificate in midwifery—not being insisted upon. Ante-natal care, however, and clinics for expectant mothers, form such an important and hopeful part of the effort to eradicate disease and rear a healthy people that good midwifery training is essential.

The third certificate recommended by the Local Government Board—that of a sanitary inspector—is also important, a practical knowledge of sanitation being part of the health visitor's daily needs.

### Duties and Offices.

It is difficult in a small space to give a useful account of the duties a woman in the public health service may be called upon to perform. Some local authorities expect her to combine in her single person the duties of district nurse and midwife, health visitor, sanitary and factory inspector, measles nurse, tuberculosis visitor, and school nurse and attendance officer! Others are less adventurous, and combine a few only of these offices.

In large urban areas, where the public health service is well established, the tendency is to divide the work into three or four departments, such as health visiting, school nursing, clinic nursing, sanitary and workshop inspection, and measles nursing. Where the staff is large enough the health visitors are responsible for the care of all the children under school age, in addition to the mothers and infants.

On reaching the age of five the children pass into the care of the Education Committee, the school nurses being responsible, under the school's medical officer, for their medical inspection, and the treatment of minor ailments. Attendance at the dental clinic is also part of the school nurse's duty. In small areas the duties of health visitor and school nurse are commonly combined, but a strong effort is now being made in the interests of the babies for the appointment of whole-time health visitors, at least in towns. Sanitary inspectors undertake inspection of workshops and factories employing women, café and restaurant kitchens, bakehouses, etc., and frequently share the infant-welfare work.

Where a whole-time clinic nurse is employed, the clinic is open daily for dressing of minor ailments, the medical officer attending one or two days a week for consultations. The health visitor should also attend, in order to know that the mother understands the doctor's advice and follows it out at home.

For the post of clinic nurse possession of the sanitary inspector's certificate is not so important, and the woman with children's or general hospital training and good midwifery experience will find herself more fitted for this work than that of health visitor.

Measles nurse, with fever training, treats and advises in severe cases nursed at home. May act as part-time health visitor between epidemics. Or she may be a district nurse lent to the sanitary authority in times of epidemic.

### The Most Useful Certificate.

It will be seen that with regard both to duties and training for public health work the "situation is still liquid," as the war correspondents say, but the following are the most useful certificates to hold:

*Central Midwives Board Certificate*, obtained in recognised maternity hospital. Fees probably between £26 and £40, according to hospital chosen. Free training may

(*Continued on page* 416.)

### PUBLIC HEALTH WORK FOR WOMEN (*Continued from page* 414.)

be had in a number of good hospitals in return for a year's services. Ordinarily the training occupies six months. Lists of recognised training institutions are obtainable from Central Midwives Board, Caxton House, Tothill Street, Westminster.

Royal Sanitary Institute Examinations.

*Certificate for Health Visitors, School Nurses, and Tuberculosis Visitors.*—In most ways this is more useful to the child-welfare worker than the sanitary inspector's certificate. Training includes infant feeding, etc., in addition to good knowledge of sanitation. Candidates are required to give evidence of nursing training, and arrangements are made for children's hospital or district nursing in connection with the sanitary science course. Cost of training varies greatly according as it is resident or non-resident. Excellent training may be obtained at such institutions as Bedford College for Women, the Royal Sanitary Institute, etc., and at the large provincial technical colleges. Fees may be as low as one or two guineas for an evening course of lectures. Day courses may be nine or ten guineas, and cost of board, etc., must be added for a residential training. Specialised courses of training are also given in connection with various baby-welfare schemes. Training given includes preparation for C.M.B. and Sanitary Institute examinations and experience at infant consultations and babies' hospitals, and the time occupied varies from one to three years.

*Sanitary Inspector's Certificate.*—Possession of this certificate gives legal right of entry into workshops and dwellings, and the inspector may compel cessation of nuisances. Preferred by some local authorities for their health visitors on this account. No nursing training is required, nor instruction in infant-welfare given.

*Maternity and Child Welfare Certificate.*—Open to women in the public health service who are certified midwives and health visitors. Special courses of lectures are given at sanitary-training centres by medical men and women for this certificate. Lists of recognised training courses are obtainable from Royal Sanitary Institute, Buckingham Palace Road, London.

Salaries and Prospects.

Salaries in the public health service are not high, ranging from £80 to £120 a year, and increasing £20 or £30 by annual increments. Travelling expenses and an allowance of £5 for uniform are paid, and in some instances a higher salary is given for the Maternity and Child Welfare Worker certificate.

Chief women sanitary inspectors and superintendent health visitors receive in general about £150 a year. With these salaries and the present high cost of living it is difficult to make any provision for the future. The interest of the work and the urgency of the need seem to be depended on to attract women to the service, but it is to be hoped their material prospects will be improved, for in this branch of work more than in most women of education and high character are needed.

For these the opportunities for leaving the world a little better than they found it are very real, either as chief of a staff, or working single-handed in a smaller area. Disappointment must often be met with, as in all pioneer work, and the duties are absorbing and often exhausting—on the home-visiting side at least. Each fresh visit paid brings a new demand on the health visitor's knowledge and sympathy—it may be only to teach the making of a pudding from a tableful of crusts; or she may be called on to dissolve a marriage, the "lady inspector" being expected to "settle it" where the magistrate fails. Questions of national insurance, soldiers' pensions, how to live on an income that is too low—or too high—and complicated moral problems, are all part of the day's work. Moral questions are not a formal part of public health work, but they cannot be put aside lightly, and the desire for wholesome and healthy living must somehow be created.

Mercifully, there is comedy as well as tragedy and dulness in public health work, and a writer on health visiting for women recommends them to cultivate "humility, reverence, and a sense of humour." A remembrance, too, that the Englishman's house is his castle, and is entered as a courtesy and not a right, will help them to do their bit to make it a home. A. R.

(*To be continued.*)

